# IKKα and IKKβ Regulation of DNA Damage-Induced Cleavage of Huntingtin

**DOI:** 10.1371/journal.pone.0005768

**Published:** 2009-06-02

**Authors:** Ali Khoshnan, Jan Ko, Simona Tescu, Patrick Brundin, Paul H. Patterson

**Affiliations:** 1 Biology Division 216-76, California Institute of Technology, Pasadena, California, United States of America; 2 Neuronal Survival Unit, Department of Experimental Medical Science, Wallenberg Neuroscience Center, Lund, Sweden; University Medical Center Groningen, Netherlands

## Abstract

**Background:**

Proteolysis of huntingtin (Htt) plays a key role in the pathogenesis of Huntington's disease (HD). However, the environmental cues and signaling pathways that regulate Htt proteolysis are poorly understood. One stimulus may be the DNA damage that accumulates in neurons over time, and the subsequent activation of signaling pathways such as those regulated by IκB kinase (IKK), which can influence neurodegeneration in HD.

**Methodology/Principal Findings:**

We asked whether DNA damage induces the proteolysis of Htt and if activation of IKK plays a role. We report that treatment of neurons with the DNA damaging agent etoposide or γ-irradiation promotes cleavage of wild type (WT) and mutant Htt, generating N-terminal fragments of 80–90 kDa. This event requires IKKβ and is suppressed by IKKα. Elevated levels of IKKα, or inhibition of IKKβ expression by a specific small hairpin RNA (shRNA) or its activity by sodium salicylate, prevents Htt proteolysis and increases neuronal resistance to DNA damage. Moreover, IKKβ phosphorylates the anti-apoptotic protein Bcl-xL, a modification known to reduce Bcl-xL levels, and activates caspases that can cleave Htt. When IKKβ expression is blocked, etoposide treatment does not decrease Bcl-xL and activation of caspases is diminished. Similar to silencing of IKKβ, increasing the level of Bcl-xL in neurons prevents etoposide-induced caspase activation and Htt proteolysis.

**Conclusions/Significance:**

These results indicate that DNA damage triggers cleavage of Htt and identify IKKβ as a prominent regulator. Moreover, IKKβ-dependent reduction of Bcl-xL is important in this process. Thus, inhibition of IKKβ may promote neuronal survival in HD as well as other DNA damage-induced neurodegenerative disorders.

## Introduction

Huntington's disease is a neurodegenerative disorder caused by expansion of a CAG repeat, which is translated into a polyglutamine (polyQ) stretch in the N-terminus of Htt protein [1]. Neurotoxicity in HD is attributed to the cleaved N-terminal fragments of mutant Htt [2]–[4]. Wild type Htt is also cleaved and inactivated by proteases, and its deletion in the central nervous system (CNS) promotes neurodegeneration and is deleterious for development [5]–[6]. The neuroprotective functions of WT Htt include inhibition of caspase-3, induction and transport of brain-derived neurotrophic factor (BDNF), and guarding against DNA damage and excitotoxicity [7]–[11].

DNA double-stranded breaks progressively accumulate in the aging brain, and elevated DNA damage is found in HD patients and HD animal models [11]–[14]. The vulnerability of neurons to DNA damage in HD is further exemplified by reduced expression of nuclear proteins such as high mobility group B 1(HMGB1), which protects against genotoxic stress [12], [15]. Elevated expression of HMGBs protects against polyQ-induced neurotoxicity in primary neurons and in a *Drosophila* polyQ model [15]. DNA damage induced by oxidative stress is also an important factor in the development of neurotoxicity and phenotypic changes in a chemical model of HD [16]. Thus, accumulation of DNA damage is a potential regulator of HD pathology. However, the mechanism for how DNA damage influences neurotoxicity in HD is not well understood. One contributing factor may be the DNA damage-induced activation of p53 and IKK signaling pathways, which have been implicated in HD neurotoxicity [14], [17]–[18].

DNA damage is a potent inducer of IKK [17]. The core complex has two kinases, IKKα and IKKβ, and a regulatory subunit, IKKγ [19]. As an activator of NF-κB, the IKK complex regulates inflammation, cytokine production and cell survival. The IKKβ subunit is the predominant kinase responsible for inflammatory responses [19]. Excessive IKKβ activity is, however, implicated in several neurodegenerative disorders, including HD, Alzheimer's disease (AD), and Parkinson's disease (PD) [18], [20]–[21]. It is relevant that HD patients have chronically elevated levels of inflammatory cytokines in the serum and CNS long before the onset of symptoms [22], implying that persistent dysregulation of IKK may occur early in the disease. In contrast, IKKα can repress IKKβ activity and reduces the production of inflammatory cytokines [23]. IKKα also has neuroprotective properties and promotes memory reconsolidation in the hippocampus [24]. Moreover, nuclear IKKα inhibits the activity of tumor suppressor p53 that is induced by DNA damage, and increases cellular resistance to genotoxic stress [25]. P53 is elevated in HD brains and reducing its activity ameliorates HD symptoms in animal models [14].

We previously showed that HD mouse models have elevated IKKβ/NF-κB in the CNS, and blocking IKKβ activity prevents degeneration of medium-sized spiny neurons caused by a toxic fragment of mutant Htt [18]. Here we report that DNA damage-induced IKKβ is an important activator of Htt proteolysis, while IKKα inhibits this event. We present evidence for a signaling network that involves phosphorylation and reduction of Bcl-xL by IKKβ, and subsequent activation of caspases, which can cleave Htt.

## Results

### DNA damage has opposite effects on IKKα and IKKβ

**Figure 1:**
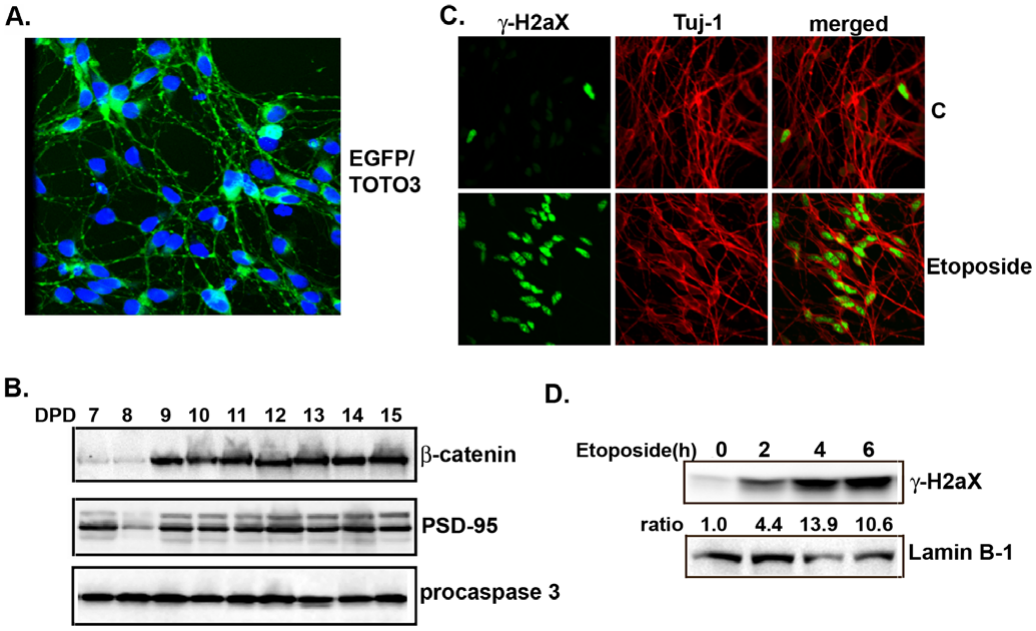
Differentiation of MESC2.10 neurons and etoposide-induced DNA damage. (A) Neuroblasts were transduced with an EGFP lentivirus. After differentiation for 9 days they were fixed and examined by a confocal microscope. TOTO-3 was used to stain the nuclei. (B) Synapse markers in MESC2.10 neurons. Extracts from differentiated neurons (DPD, days post differentiation) were examined by Western blotting for expression of β-catenin and PSD-95. Bottom panel shows Western blot analysis of lysates from neurons maintained for different time points in culture stained for caspase-3 activation. (C) γ-H2aX accumulates in the nuclei of etoposide treated MESC2.10 neuron. Differentiated neurons treated with etoposide for 4hr were fixed and stained with a rabbit anti-γ-H2aX (green). Anti-Tuj-1 was used to label the cytoplasm (red). Part (D) shows accumulation of γ-H2aX in the nuclear fraction of etoposide treated neurons over time examined by Western blotting with anti-γ-H2aX antibody. Lamin B1 was used as loading control.

In this study we used a human embryonic neuronal stem cell line (MESC2.10) isolated from the midbrain of an eight-week-old embryo [26], to characterize the signaling between DNA damage, IKKβ activation and Htt turnover. To establish the model, MESC2.10 neuroblasts were differentiated and examined for expression of neuronal markers. Upon differentiation, MESC2.10 cells acquire neuronal morphology (Fig. 1A) and express neuron-specific proteins such as PSD-95, β-catenin and the neurofilament Tuj-1 (Fig.1B, top two panels and 1C). These neurons can be maintained for more than two weeks without significant apoptosis (Fig. 1B, third panel). To induce DNA damage, we first used the topoisomerase inhibitor etoposide, which produces DNA double-stranded breaks in post-mitotic neurons [27]. The induction of DNA damage in MESC2.10 neurons was confirmed by nuclear staining of phosphorylated histone H2aX (γ-H2aX), a surrogate marker of DNA damage (Figs. 1C and 1D) [28]. We used an acute etoposide treatment of 6 hr to avoid neuronal death, which occurs after prolonged incubation [27].

**Figure 2:**
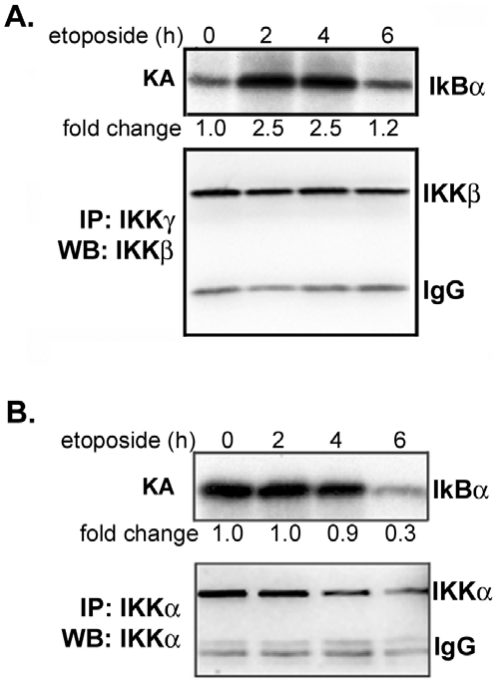
Etoposide promotes IKKβand inhibits IKKα. (A) Etoposide activates IKKβ. IKK complexes were immunoprecipitated with anti-IKKγ antibody coupled to protein G agarose beads and assayed for kinase activity using GST-IκBα and ^32^P-γ-ATP. Products were examined by SDS-PAGE followed by autoradiography. The top panel shows kinase activity (KA) and the lower panel shows a Western blot for IKKβ of similar immunoprecipitated complexes. (B) IKKα is constitutively active in MESC2.10 neurons. Lysates were first treated with a combination of anti-IKKβ and IKKγ antibodies coupled to agarose beads to deplete IKKγ/IKKβ/IKKα complex. IKKα complexes were then immunoprecipitated with anti-IKKα antibody conjugated to protein G agarose beads and assayed for kinase activity as described in part A. The top panel shows IKKα activity and the bottom panel shows the Western blot for IKKα. Fold changes of IKK activity were quantified by measuring the band intensity using Image J, and compared to non-treated neurons.

To determine if DNA damage activates IKK in neurons we first performed *in vitro* kinase assays using recombinant IκBα as the substrate for IKKβ complexes immunoprecipitated from the extracts of etoposide-treated neurons. We find that IKKβ is activated 2 hrs after etoposide treatment and remains active for up to 4 hrs (Fig. 2A top panel). These results are consistent with the effects of etoposide on IKKβ in non-neuronal cells [17]. We were also interested in whether DNA damage influences IKKα activity, since this subunit appears to play a prominent role in survival and neuronal plasticity [24]–[25]. To obtain IKKα complexes devoid of IKKβ, neuronal extracts were first depleted by prior incubation with anti-IKKγ and -IKKβ antibodies, and IKKα complexes were subsequently immunoprecipitated. IKKα activity is routinely tested *in vitro* by phosphorylation of IκBα, although *in vivo* this phosphorylation is predominantly performed by IKKβ [19]. Interestingly, MESC2.10 neurons display constitutive IKKα activity (Fig. 2B, top lane 1). MESC2.10 neurons are grown in complex medium with several growth factors, some of which might be responsible for activation of IKKα. Longer treatment with etoposide, however, reduces IKKα activity (Fig. 2B, lanes 4). We also observe a decrease in the level of IKKα protein, which may contribute to the low IKKα activity (Fig. 2B, bottom panel). Thus, induction of DNA damage has opposite effects on the activity of IKKα and IKKβ in neurons.

### DNA damage-induced proteolysis of Htt is regulated by IKKs

**Figure 3:**
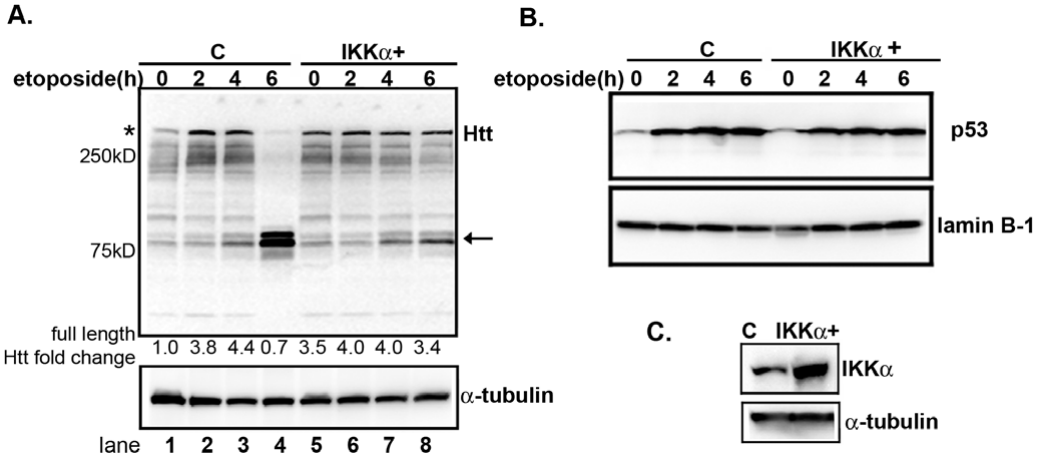
Etoposide promotes Htt proteolysis and that is inhibited by elevated IKKα expression. (A) EGFP and IKKα-transduced neurons were treated with 10 µM of etoposide for the indicated times. Extracts were examined for Htt by Western blotting. The top panel shows staining with anti-Htt (mAb 2166) antibody. The asterisk indicates full-length endogenous Htt and the arrow shows the cleaved products. The second panel shows staining for tubulin. Fold changes for full-length Htt levels were obtained by measuring the band intensity in each lane, normalized to tubulin and compared to non-treated control. (B) p53 accumulates in the nucleus of etoposide treated neurons. Nuclear extracts from MESC2.10 neurons were examined for the presence p53 by Western blotting. Lanes 1–4 are nuclear extracts from MESC2.10 neurons with EGFP and lanes 5–8 are from neurons that were transduced with IKKα lentiviruses (Fig. 1C). Staining with anti-lamin B1 was used to ensure equal loading (bottom panel). (C) Western blot analysis of IKKα levels in the control and IKKα-expressing neurons. MESC2.10 neuroblasts were transduced with an IKKα recombinant lentivirus and differentiated as described in M&M. EGFP lentivirus was used as a control. Top panel shows the Western blot for IKKα and bottom panel is staining of the same blot for tubulin.

Induction of p53 by DNA damaging agents promotes the expression of Htt [29]. Consistent with these observations, we find that treatment of MESC2.10 neurons with etoposide increases the level of endogenous, full-length Htt ∼4-fold (Fig. 3A, top panel, asterisk, lanes 1–3). The elevation of Htt overlaps in time with accumulation of nuclear p53 (Fig. 3B, top panel, lanes 1–4). Longer exposure of neurons to etoposide induces proteolysis of endogenous Htt, however, generating N-terminal fragments of ∼80 kDa (Fig. 3A, top panel, lane 4, arrow). These data suggest that accumulation of DNA damage in neurons activates proteolytic enzymes that can cleave Htt.

To confirm that Htt cleavage is induced by DNA damage and not other, secondary effects of etopoisde, we examined whether DNA damage induced by γ-irradiation of neurons could have a similar effect on Htt cleavage. As expected, γ-irradiation also generates an Htt fragment similar in size to that produced by etoposide treatment (Fig. S1). Interestingly, induction of Htt proteolysis by γ-irradiation occurs faster than etoposide treatment and is prominent by 4 hr post-irradiation, whereas maximal Htt proteolysis induced by etoposide requires ∼6 hr. The difference maybe due to rapid induction of double stranded DNA breaks by irradiation.

Since etoposide treatment reduces the level and activity of IKKα (Fig. 2B), we examined whether increasing its level could rescue the effects of DNA damage on Htt protein. MESC2.10 neurons were transduced with an IKKα lentivirus, which increases the level of IKKα by ∼3-fold (Fig. 3C). Indeed, this enables the IKKα^+^ neurons to resist etoposide-induced Htt proteolysis (Fig. 3A, top panel, lanes 5–8). IKKα^+^ neurons display a higher basal level of full-length WT Htt than controls (Fig. 3A, compare lanes 1 and 5), and accumulation of p53 in IKKα+ neurons (Fig. 3B, top panel, lanes 5–8) has no additional effect on Htt levels. The lack of further Htt elevation in IKKα^+^ neurons may be due to inhibition of p53 transcriptional activity by elevated IKKα [25]. While the interaction between p53, IKKα and Htt expression remains to be explored in detail, these findings support a protective role for IKKα in reducing proteolysis of endogenous, WT Htt in neurons with DNA damage.

**Figure 4:**
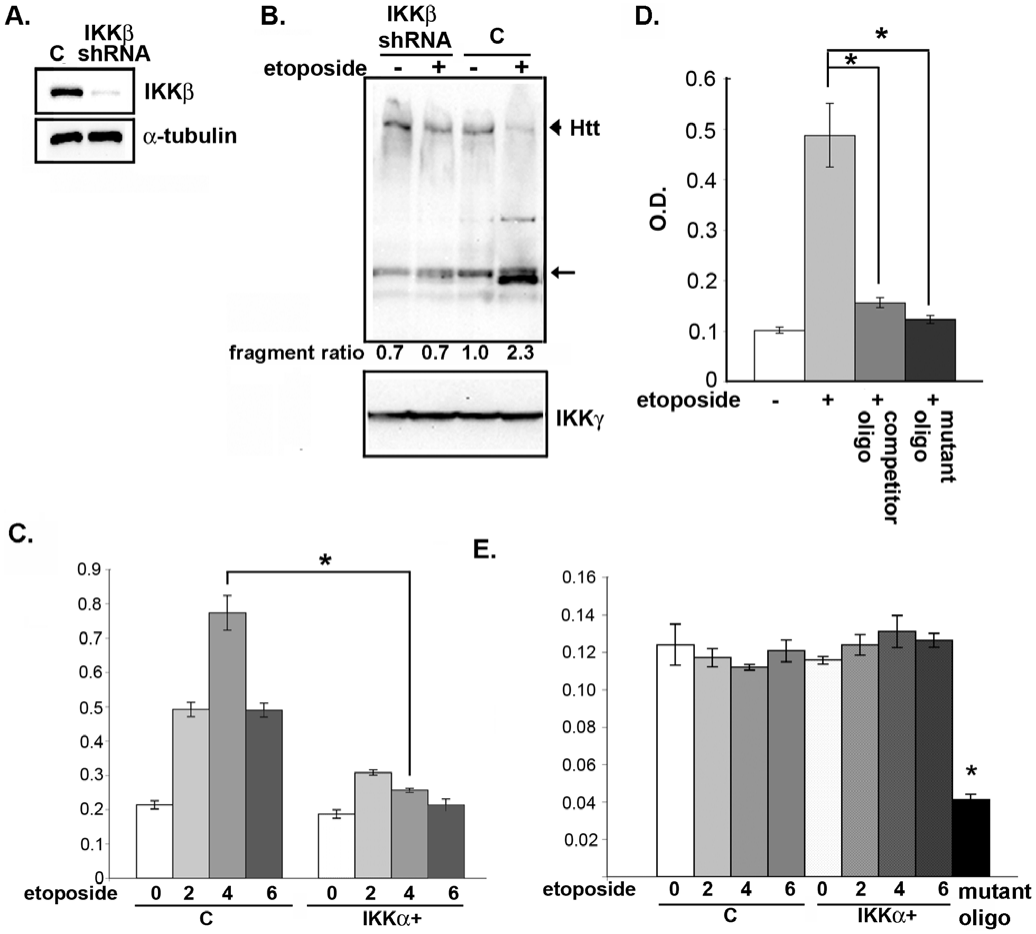
Inhibition of IKKβ prevents proteolysis of Htt induced by etoposide. (A) MESC2.10 neurons were transduced with a control or lentivirus expressing specific anti-IKKβ shRNA. The level of IKKβ protein was examined by Western blotting with an anti-IKKβ antibody. Bottom panel shows staining of same blot for α-tubulin. (B) Inhibition of etoposide-induced Htt cleavage by silencing of IKKβ. Control or MESC2.10 neurons with silenced IKKβ were treated with etoposide for 6 hrs and examined for Htt cleavage (lanes 1 and 2) as described in Fig. 3A. Arrowhead indicates position of full length Htt, and the arrow shows the position of the major cleaved product. The second panel shows IKKγ levels used as a loading control. (C) DNA binding activity of P65 NF-κB is increased by etoposide and is suppressed by IKKα. Binding to consensus NF-κB oligonucleotides and detection was described in M&M. Lanes 1–4 show p65 binding from nuclear extracts of control and lanes 5–8 are from neurons transduced with a lentivirus expressing IKKα (Fig. 3C). Bars indicate S.E.M. and asterisk shows significant difference between control and IKKα+ neurons treated with etoposide for 4 hr, P <0.01 using a student's t test. (D) Competition of etoposide-induced p65 NF-κB binding by consensus oligonucleotides. Nuclear extracts of etoposide treated neurons were pre-incubated with 100 ng of competitor NF-κB oligonucleotides (Clontech) on ice for 1 hr (Column 3) before treatment with etoposide for 6 hrs. Wells with mutated NF-κB DNA oligonucleotides were used to ensure specificity of the binding (column 4). (E) Binding of p52 NF-κB to consensus oligonucleotides is not changed by DNA damage. Experiments were similar to in part C, except binding was examined for p52. Bars indicate S.E.M. and asterisks show significant difference in binding between samples without or with the competitor oligonucleotides or with p65 binding to mutated NF-κB oligonucleotides, P value <0.01, using a student's t test.

In contrast, IKKβ activity is induced by DNA damage (Fig. 2A). Thus, we hypothesized that reducing its level may be protective. Towards this end, we silenced IKKβ expression with a specific, anti-IKKβ small hairpin RNA (shRNA) expressed from a lentivirus (Fig. 4A), and treated the neurons with etoposide. We find that, similar to the effect of elevating IKKα, silencing IKKβ expression reduces the proteolysis of Htt (Fig. 4B). Silencing of IKKβ expression or inhibition of its kinase activity by sodium salicylate [30] also blocks Htt cleavage induced by γ-irradiation (Fig. S1, lanes 8 and 5, respectively). Thus, IKKβ activation by DNA damage promotes Htt cleavage, and increasing IKKα or reducing IKKβ blocks this event.

It is relevant that etoposide-induced IKKβ activates p65 NF-κB DNA binding in MESC2.10 neurons (Figs. 4C and D). Interestingly, the DNA binding activity of p65 is significantly reduced in neurons with elevated IKKα (Fig. 4C). While IKKα is known to inhibit IKKβ/NF-κB in immune cells [23], this has not been reported for post-mitotic neurons. Therefore, the protective effects of IKKα in response to DNA damage may include inhibition of IKKβ activity. However, inhibitors of NF-κB do not influence Htt proteolysis, suggesting that IKKβ regulation of Htt proteolysis is NF-κB independent (see below). Etoposide has no effect on the activation of p52 NF-κB in MESC2.10 neurons (Fig. 4D).

### IKKs influence the DNA damage-induced activation of pro-apoptotic caspases

**Figure 5:**
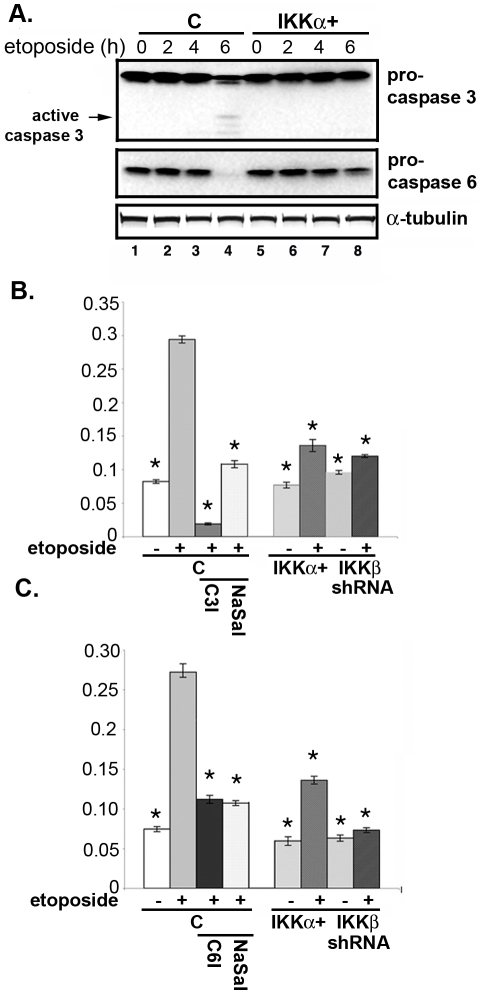
IKKs influence etoposide-induced activation of caspases. (A) Activation of caspase-3 and caspase-6. MESC2.10 neurons were treated with etoposide as in figure 3A and examined for the levels of procaspase-3 (top panel) or procaspses-6 (middle panel) by Western blotting. Arrow shows the cleaved products of procaspase-3. (B and C) Caspase-3 (B) and caspase-6 (C) activities are shown in MESC2.10 neuronal lysates. For the specific inhibitors neurons were first pretreated with 20 µM of Ac-DEVD-CHO, caspase-3 inhibitor (C3I) or 20 µM of Ac-VEID-CHO, caspase-6 inhibitor (C6I), or 5 mg/ml of sodium salicylate (NaSal) one hr prior to etoposide treatment for 6 hrs. Extracts were incubated with either caspase-3 substrate (DEVD conjugated to p-nitroanaline) or caspase-6 substrate (VEID conjugated to p-nitroanline) in a 96 well plate at 37°C for 1 hr. Enzyme activities for caspase-3 (B) or caspase-6 (C) were measured in a microplate reader. Results are shown as relative enzyme activity and represent averages of three experiments. Bars indicate S.E.M. and asterisk shows significant difference from etoposide treated control neurons (column 2), p<0.01, using a student's t test.

Several caspases, including caspase-3 and -6, are known to cleave Htt between amino acids 500–600 generating fragments similar in size to those observed in our DNA damage paradigm (Fig. 3A) [2]–[5]. To better understand the role of the IKKs in etoposide-induced Htt cleavage, we measured the activity of the caspases. Both procaspase-3 and -6 levels are reduced after 6 hrs of etoposide treatment (Fig. 5A, lane 4), which coincides with the timing of Htt proteolysis (Fig. 3A, lane 4). Consistent with the reduction of procaspases, the extracts of etoposide-treated neurons display elevated caspase-3 and caspase-6 activities and are blocked by specific caspase inhibitors (Figs. 5B and 5C, columns 2 and 3, respectively). On the other hand, neurons with elevated IKKα resist activation of procaspase-3 and procaspase-6 (Fig. 5A, compare lanes 4 and 8). Moreover, extracts of etoposide-treated IKKα^+^ neurons contain reduced caspase-3 and caspase-6 activity (Figs. 5B and 5C, column 6, respectively). Blocking IKKβ activity with sodium salicylate, a potent inhibitor of IKKβ [30], or silencing its expression by shRNA also lowers the etoposide-induced activation of caspases (Figs. 5B and 5C, columns 4 and 8, respectively). Thus, the opposite effects of IKKα and IKKβ on caspase activation in the context of DNA damage may underlie their differential effects on Htt cleavage.

### DNA damage-induced IKKβ regulates Bcl-xL

**Figure 6:**
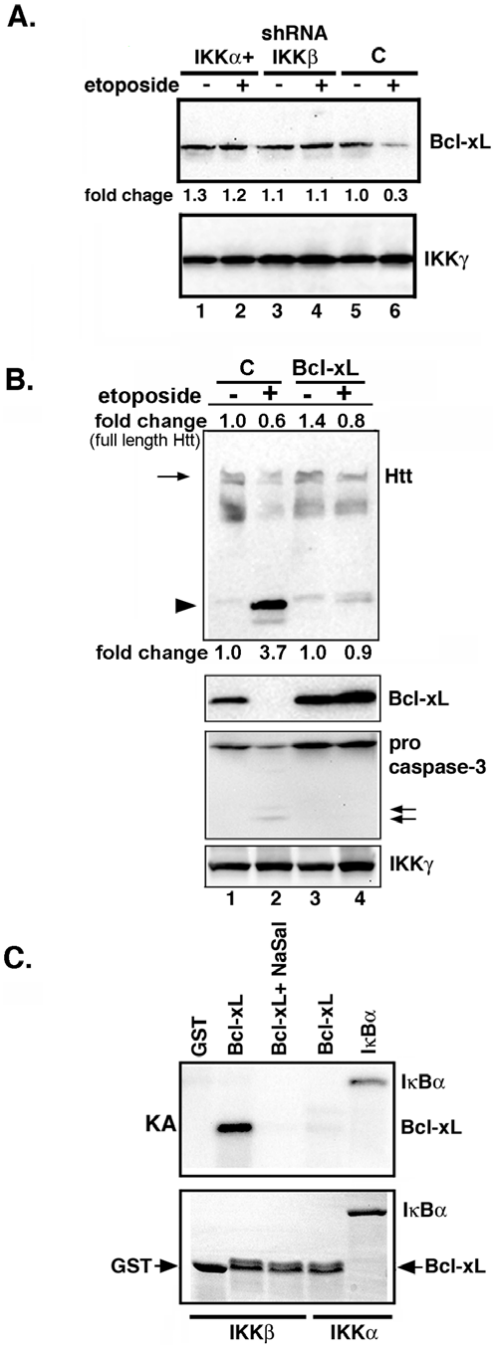
Etoposide promotes reduction of Bcl-xL. (A) Extracts of control and etoposide-treated MESC2.10 neurons were examined for Bcl-xL by Western blotting. Neurons were treated with etoposide for 6 hrs. Top panel shows staining for Bcl-xL and the bottom panel indicates IKKγ as a loading control. Fold changes were normalized to the intensity of loading control, and compared to that of untreated control neurons (lane 5). (B) Bcl-xL expression prevents Htt proteolysis by etoposide. MESC2.10 neuroblasts were transduced with a lentivirus expressing Bcl-xL (Lanes 3 and 4) and treated with etoposide for 6 hrs. EGFP-lentivirus was used a control (C). Top panel Western shows blotting for Htt. Arrow indicates the full-length Htt and the arrowhead shows the cleaved Htt products. Second and third panels show staining for Bcl-xL and pro-caspase-3, respectively. IKKγ levels were used as a loading control. (C) IKKβ phosphorylates Bcl-xL. Active recombinant IKKα or IKKβ were tested for the ability to phosphorylate Bcl-xL. The kinase assay was performed as described in the M&M with recombinant Bcl-xL as a substrate. Products were visualized by autoradiography. IκBα was used as a positive control substrate for IKKα. The top panel shows the kinase product (KA) and bottom panel shows the SDS-PAGE and coomassie-blue staining of the substrates use in kinase assays.

Activation of caspases can be triggered by several mechanisms, including reduction in Bcl-xL [31]. The prosurvival protein Bcl-xL, which is abundant in the CNS, has recently been implicated in Htt proteolysis in a chemical model of HD [32]–[33]. We find that the level of intact Bcl-xL is reduced in extracts of neurons treated with etoposide (Fig. 6A, lane 6). In contrast, etoposide treatment does not affect Bcl-xL in neurons with elevated IKKα or reduced IKKβ expression (Fig. 6A, lanes 2 and 4, respectively). To confirm that Bcl-xL is important for blocking of DNA damage-induced Htt proteolysis, we increased its expression in MESC2.10 neurons using a recombinant lentivirus (Fig. 6B, second panel, lanes 3 and 4). As predicted, elevated Bcl-xL prevents Htt proteolysis by DNA damage and this overlaps in time with prevention of caspase-3 activation (Fig. 6B first and third panels, respectively). Although Bcl-xL expression prevents DNA damage-induced Htt cleavage, some reduction in the level of full-length Htt is also observed. Whether etoposide treatment induces production of other Htt fragments that are not detected by this anti-Htt antibody and not protected by Bcl-xL, remains to be investigated. Overall, these studies indicate that Bcl-xL level is a critical component of caspase-mediated Htt proteolysis induced by DNA damage, and is likely influenced by IKKβ.

The level of Bcl-xL mRNA in MESC2.10 neurons is not affected by etoposide (data not shown), indicating that Bcl-xL reduction is likely due to enhanced protein turnover. Although phosphorylation-induced degradation of Bcl-xL in the presence of genotoxic agents is a necessary step for induction of apoptosis, it is unclear which kinase(s) mediates this event [31]. We tested the possibility that activated IKKβ phosphorylates Bcl-xL. To avoid contamination with other neuronal kinases that may co-immunoprecipitate with IKKβ, we used recombinant IKKs for *in vitro* kinase assays with Bcl-xL as the substrate. Indeed, IKKβ phosphorylates Bcl-xL (Fig. 6C, top panel), and this is specific since inhibition of IKKβ by sodium salicylate prevents this reaction, and IKKα does not phosphorylate Bcl-xL. Thus, IKKβ is a novel kinase that modifies Bcl-xL and reduces its level in stressed, post-mitotic neurons. This is consistent with the unchanged levels of Bcl-xL in etoposide-treated neurons in which IKKβ expression has been silenced (Fig. 6A).

### DNA damage-induced Htt proteolysis is polyQ-independent

**Figure 7:**
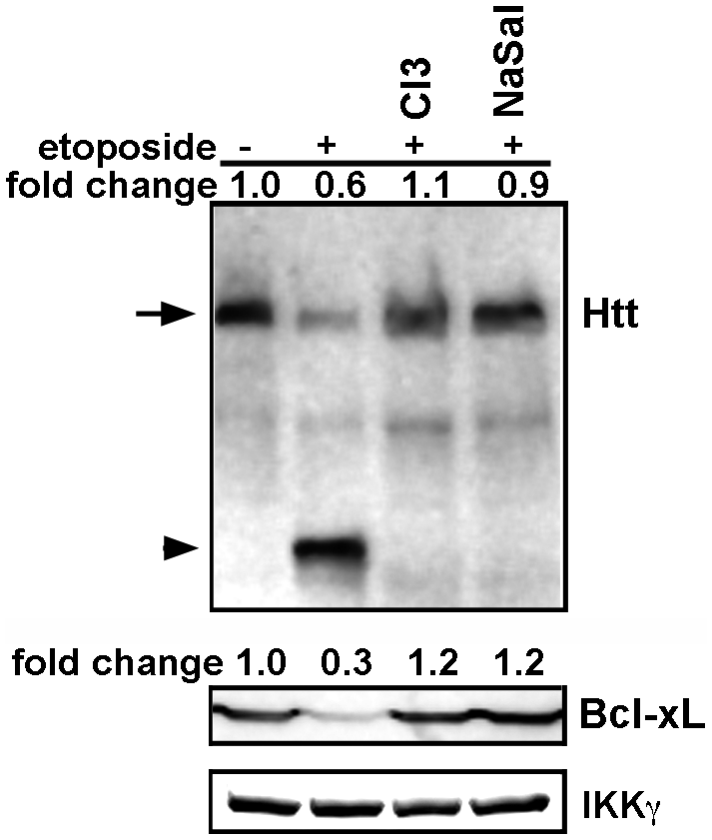
Etoposide induces proteolysis of endogenous mutant Htt in neurons striatal (Hdh^Q111/Q111^) neurons. The caspase-3 inhibitor (C3I, 20 µM) and sodium salicylate (NaSal, 5 mg/ml) were added 1 hr prior to the addition of 10 µM etoposide for 6 hrs. Processing of samples was as described in figure 3A. Top panel shows Western blot analysis of lysates for Htt. Arrow indicates the full-length Htt and the arrowhead shows the cleaved products (∼90 kDa). The second panel shows the level of Bcl-xL. IKKγ was used as loading control.

Generation of N-terminal fragments of mutant Htt is thought to initiate neurotoxicity, culminating in HD [2]–[4]. We tested whether DNA damage also promotes the proteolysis of full-length mutant Htt. In a striatal neuronal line obtained from HD knock-in mice (Hdh^Q111/Q111^) [34], we find that etoposide treatment also promotes cleavage of full-length, mutant Htt protein (Fig. 7, top panel, lane 2). This proteolysis is blocked by inhibition of caspase-3 or IKKβ activity (Fig. 7 top panel, lanes 3 and 4). Moreover, cleavage of Htt overlaps in time with reduction of Bcl-xL, which is prevented by inhibition of IKKβ with sodium salicylate (Fig. 7, second panel). Taken together, these results indicate that, in post-mitotic neurons, DNA damage-activated IKKβ facilitates Htt proteolysis indirectly by promoting Bcl-xL turnover and activating a caspase pathway. Thus, in the context of neuronal DNA damage, IKKβ activation is detrimental and its inhibition may be protective in HD and potentially in other neurodegenerative disorders where DNA damage plays a role.

## Discussion

**Figure 8:**
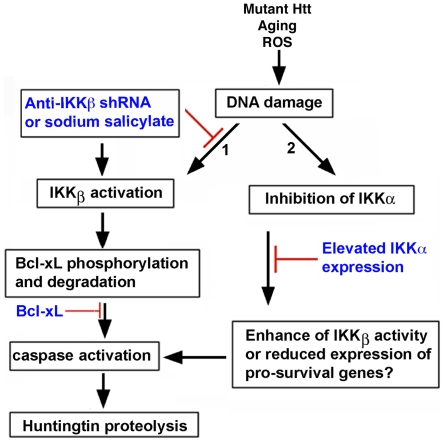
A schematic diagram showing a potential signaling pathway for IKKβ-mediated Htt proteolysis in MESC2.10 neurons. (A) DNA damage activates IKKβ, which can phosphorylate Bcl-xL and enhance its degradation (arrow 1). Reduction of Bcl-xL levels triggers the activation of caspases, which cleaves Htt. IKKβ inhibition block degradation of Bcl-xL, caspases activation, and proteolysis of Htt. Similar to the inhibition of IKKβ, elevation of Bcl-xL also prevents caspase activation and Htt proteolysis. On the other hand, etoposide treatment reduces the activity of IKKα (arrow 2). This may enhance IKKβ activation and/or block expression of neuroprotective proteins that are essential for interfering with caspase activation and maintaining Htt levels. Elevated IKKα expressed from a lentivirus overcomes these deficiencies and prevents Htt proteolysis.

The novel aspects of present work are the triggering of Htt proteolysis by DNA damage and the identification of IKKβ as a prominent regulator of enzymes known to cleave Htt. We propose that in post-mitotic neurons, IKKβ activation by DNA damage promotes Htt cleavage by influencing Bcl-xL and a caspase pathway. Conversely, our data support a protective role for IKKα, whose expression in neurons prevents DNA damage-induced Htt proteolysis (Fig. 8).

Although acute IKKβ activation is known to be predominantly pro-survival, under certain circumstances it promotes apoptosis and neurodegeneration [20], [35]. For example, conditional deletion of IKKβ delays apoptosis by inhibiting caspase-3 in regressing mammary epithelial cells [35]. Aberrant activation of IKKβ has also been implicated in neurotoxicity in HD, PD, AD, multiple sclerosis, and ischemia-induced neuronal death [18], [21], [36]–[38]. Our data indicate that activation of IKKβ by DNA damage may injure neurons by increasing the turnover of WT Htt. It is notable that both WT and mutant Htt are cleaved in our DNA damage paradigm. Neurons in HD brains, which have WT and mutant copies of Htt, may therefore be susceptible to DNA-induced damage on both counts. In addition to loss of WT Htt function, cleavage of the mutant Htt could also generate toxic N-terminal fragments that are amyloidogenic [4]. Thus, DNA damage-induced IKKβ in the HD brain may set in motion one of the earliest events in HD pathology. Other insults such as chronic exposure to pro-inflammatory cytokines, which are elevated in the plasma and brain tissue of pre-symptomatic HD patients, may also cause persistent activation of IKKβ in neurons and further exacerbate the cleavage of Htt [19], [22]. A potential source for these cytokines is the activated microglia that are detected in various parts of HD brains, including striatum and hypothalamus [39], [40]. It is interesting that deletion of IKKβ in microglia reduces neurodegeneration in a chemical model of HD by suppressing the expression of pro-inflammatory cytokines [41]. While the role of cytokines in IKKβ-dependent Htt proteolysis remains to be characterized, we can speculate that activation of IKKβ in neurons may trigger Htt proteolysis cell autonomously, as in the induction of DNA damage, or non-autonomously by chronic exposure to pro-inflammatory cytokines produced by microglia (41). It is also of interest that inhibitors of IKKβ reduce proteolysis of amyloid precursor protein (APP) and subsequent Aβ42 production [38]. The regulation of amyloidogenic proteins such as Aβ and N-terminal Htt fragments by activated IKKβ is an intriguing area for future work.

In contrast to IKKβ, IKKα promotes neuronal survival and prevents Htt proteolysis. This could simply result from suppression of IKKβ activity by IKKα (Fig. 4C) [23]. However, IKKα has several other neuroprotective properties that could be important. For example, IKKα activates CREB binding protein (CBP), a transcriptional co-activator of many neuronal signaling pathways including BDNF expression [25], [42]. Enhancement of the histone acetyltransferase activity of CBP is known to reduce the toxicity of mutant Htt [43]. IKKα also inhibits the transcriptional activity of p53, which has been implicated in HD pathogenesis, and promotes neuronal plasticity and memory formation in the hippocampus [14], [24]–[25]. Thus, IKKα may prevent Htt proteolysis independent of IKKβ by modulating expression and/or activation of gene products that antagonize the toxic effects of DNA damage. Further examination of IKKα using gene therapy or compounds that selectively enhance its functions *in vivo* is worthy of investigation in HD animal models.

The mechanism of Htt proteolysis is complex and probably involves integration of multiple signaling pathways. Our results identify Bcl-xL as a potential mediator of DNA damage-induced Htt proteolysis (Fig. 6). Bcl-xL participates in neuronal survival, and its degradation results in synaptic degeneration [32]. Our findings indicate that DNA damage reduces Bcl-xL levels in an IKKβ-dependent manner. A role for IKKβ is supported by the inability of etoposide to reduce Bcl-xL in neurons expressing an shRNA targeting IKKβ, or following pretreatment with sodium salicylate, a potent inhibitor of IKKβ (Figs. 6A and 7). We propose that IKKβ reduces Bcl-xL levels by phosphorylation (Fig. 6C), a modification known to promote Bcl-xL degradation [31]. Phosphorylation-dependent reduction of Bcl-xL is suggested to play a role in spinal cord neuronal injury and is implicated in Htt proteolysis in the striatum of 3-nitropropionic acid injected mice [33], [44]. Thus, reduction of Bcl-xL may be the signal that leads to activation of caspases that target Htt (Fig. 8). Inhibition of caspase-3 activation and Htt proteolysis in neurons with elevated Bcl-xL further supports this notion (Fig. 6B).

Environmental factors appear to play a role in the development of HD, since the age of onset is variable among patients with similar polyQ expansions [45]. Accumulation of DNA damage as well as activation of modifiers that influence Htt proteolysis could impact disease progression. Our data indicate that IKKβ induced by DNA damage in neurons can be very significant, and inhibition of IKKβ can prevent depletion of WT Htt as well as the production of toxic N-terminal fragments of mutant Htt. It is noteworthy that IKKβ is itself activated by N-terminal fragments of mutant Htt [18]. Thus, Htt proteolysis and IKKβ activation can form a toxic feedback loop that could promote neurodegeneration. This cycle can be influenced by cytokines that are induced by IKKβ activation and which are elevated in HD patients [22]. IKKα, on the other hand, may act as a brake to suppress excessive IKKβ activation and reduce neurotoxicity (Fig. 8). Thus, the ratio of IKKα to IKKβ activity may be a determinant of Htt proteolysis in stressed neurons. Identification of the IKKs as regulators of Htt proteolysis offers novel targets to search for molecules that prevent one of the earliest events in HD pathogenesis.

## Materials and Methods

### Antibodies and reagents

Anti-Htt (mAb 2166), and PSD-95 antibodies were purchased from Millipore (Temecula, CA). Antibodies recognizing IKKγ, IKKβ, caspase-3 and caspase 6 were purchased from Cell Signaling Technology (Danvers, MA). Anti-IKKα was purchased from BD Biosciences (San Diego, CA). Anti-Bcl-xL and anti-p53 antibodies were from Santa Cruz Biotechnologies (Santa Cruz, CA). Anti-Tuj-1 antibody was purchased from Covance (Berkeley, CA). Cell fractionation and ECL detection kits including HRP-conjugated secondary antibodies were from PIERCE Biotechnology (Rockford, IL). Anti-γ-H2AX antibody, caspase-3 and caspase-6 activity kits, and recombinant Bcl-xL protein were obtained from R&D systems (Minneapolis, MN). Anti-laminB1 antibody, DMEM/F12, N-2 and B-27 media supplements were purchased from Invitrogen (Carlsbad, CA). Fibroblast-growth factor-2 (FGF-2) was obtained from Promega (Madison, WI). Sodium salicylate, etoposide, and caspase-3 and caspase-6 inhibitors (Ac-DEVD-CHO and Ac-VEID-CHO, respectively), and anti-tubulin antibody were purchased from Sigma/Aldrich (St. Louis, MO). Recombinant active IKKα and IKKβ were purchased from Upstate Biotechnologies (Lake Placid, NY). Consensus NF-κB oligonucleotides for p65 and p52 coated onto 96 wells and the corresponding antibodies for binding detection (TransFactor NF-κB p65 and p52) were purchased from Clontech (Mountain View, CA)

### Generation of MESC2.10 human neurons

The methods for generation and differentiation of MESC2.10 human neuroblasts have been reported previously [26]. Briefly, neuroblasts obtained from an 8 week-old human embryo were transduced with a retrovirus encoding a tetracycline-regulated v-myc to promote proliferation. These neuroblasts were grown on poly-lysine and laminin coated plates in DMEM/F12 in the presence N2 and B-27 neuronal supplements (Invitrogen) and 20 ng/ml FGF-2 (Promega). To differentiate MESC2.10 cells, proliferation medium was replaced DMEM/F12 medium containing 2 µg/ml of doxycycline and 5 µM cAMP.

Mouse striatal neuronal precursors, Hdh^Q111/Q111^, obtained from homozygous knock-in mice were propagated as described [34]. Differentiation was carried out in DMEM/F12 medium containing N2 and B-27 supplements as described for MESC2.10 neurons.

### Etoposide treatment, cell fractionation and Western blotting

MESC2.10 cells, differentiated for 9 days, were treated with 10 µM etoposide for the periods indicated in each figure. Neurons were harvested and separated into cytoplasmic and nuclear fractions using the NE-PER kit (PIERCE) according to instructions. For most experiments we used ∼120 µg of lysate for SDS-PAGE and Western blotting with the indicated antibodies. Reactive bands in Western blots were detected by enhanced chemiluminescence (ECL) using a gel documentation system. Sodium salicylate (5 mg/ml), caspase-3 inhibitor (Ac-DEVD-CHO) and casapse-6 inhibitor (Ac-VEID-CHO) were added at 20 µM 1 hr prior to etoposide treatment. To examine the effects of etoposide on mutant Htt differentiated mouse striatal cells (Hdh^Q111/Q111^) were treated with etoposide and analyzed as described for MESC2.10 cells.

### Immunochemistry

Differentiated neurons on coverslips were treated with 10 µM etoposide for 4 hr. Cells were fixed and stained with a rabbit antibody that specifically recognizes H2aX phosphorylated at Ser 139 (γ-H2aX) (1∶500) (R&D systems). Anti-Tuj-1 was used to label the cytoplasm (1∶1000). Goat anti-rabbit conjugated to FITC (green) and goat anti-mouse conjugated to rhodamine (red) was used as secondary antibodies. Pictures were captured with a confocal microscope.

### Lentivirus production

IKKα was cloned into the lentiviral FUGW vector under the control of a ubiquitin promoter [46]. An EGFP-lentivirus was used as a control. The Bcl-xL cDNA was cloned from MESC2.10 neurons by RT-PCR using standard procedures and its identity confirmed by sequencing. The cDNA was subsequently inserted into FUGW lentiviral vector. The shRNAs for IKKβ cloned in a lentiviral backbone was purchased from Open Biosystems (Huntsville, AL). Lentiviruses were produced by transfection of 293 cells using calcium phosphate precipitation [46]. Supernatants of virus-producing cells were harvested 48 hr post-transfection and concentrated on Amicon Ultra columns (Millipore, MA). An EGFP virus was used as a control to monitor viral titer. A multiplicity of infection of 4:1 was used to infect MESC2.10 neuroblasts. Expression of IKKα, Bcl-xL and reduction of IKKβ were determined by Western blotting.

### Kinase assay

IKKβ activity was assayed as described previously [18]. To determine IKKα activity, lyastes were first depleted of IKKβ complexes using 4 µg of anti-IKKγ and anti-IKKβ antibodies coupled to protein G. The depleted lysates were used to isolate IKKα complexes with 2 µg of anti-IKKα antibody. GST-IκBα was used as a substrate to measure kinase activity. To examine whether IKKs phosphorylate Bcl-xL, 0.5 µg of either IKKα or IKKβ were incubated with 1 µg of full-length Bcl-xL in the presence of ^32^P-γ-ATP for 30 min at 30°C. GST protein was used as a negative control. All kinase products were examined by SDS-PAGE and autoradiography.

### Assay of NF-κB binding to consensus DNA oligonucleotides

Nuclear extracts for control or etoposide-treated MESC2.10 neurons were obtained using the cell fractionation kit from pierce according to the instructions provided. Fifty µg of each nuclear extract was incubated for an hour at RT on 96 well plates (Clonetech, Mountain View, CA) coated with consensus NF-κB oligonucleotides (p65 or p52). Mutated NF-κB oligonucleotides were used to confirm binding specificity. For competition assays, nuclear extracts were pre-incubated with NF-κB oligonucleotides for 1 hr on ice and then added to the coated wells. After washing, each well was incubated with anti-p65 or anti-p52 antibodies for 30 min at 37°C. Wells were washed as recommended by the manufacturer (Clontech), followed by incubation with a secondary antibody conjugated to HRP. TMB (3, 3′, 5, 5′-tetramethylbenzidine) was added for 10 min. Binding was measured in a microplate reader at 655 nm.

### Caspase assay

To measure the activity of caspase-3 or -6, the colorimetric assay kits from R&D Systems were used according to manufacturer's instructions. Briefly, MESC2.10 neurons were pre-incubated with caspase-3 inhibitor (Ac-DEVD-CHO), casapse-6 inhibitor (Ac-VEID-CHO) or sodium salicylate (5 mg/ml) 1 hr prior to etoposide treatment, which was for an additional 6 hrs. Cells were lysed as instructed. Fifty µg of each lysate was incubated with either caspase-3 substrate (DEVD conjugated to p-nitroanaline) or caspase-6 substrate (VEID conjugated to p-nitronalaine) in a 96 well plate at 37°C for 1 hr. Enzyme activities for caspase-3 or -6 were measured with a microplate reader at 405 nm. Results are shown as relative enzyme activity and represent average of three experiments.

## Supporting Information

Figure S1 γ-irradiation induced DNA damage promotes Htt cleavage in MESC2.10 neurons. γ-irradiation was carried out using a MARK-I γ-irradiator with a 137Cs source at a specific dose rate of 1.22 Gy/min. Cells were irradiated with 5 Gy and further incubated for the indicated time. Sodium salicylate (NaSal, lane 5) was added at a concentration of 5 mg/ml 1 hr prior to irradiation and incubated for 6 hr. Etoposide treatment (10 µM) was used as a positive control and was carried out for 6 hr (lane 6). Lanes 7 and 8 represent MESC2.10 neurons transduced with an IKKβ shRNA lentivirus (Fig. 4A) and irradiated with 5 Gy for 6 hr. The asterisk shows full-length Htt and the arrow indicates the cleaved products. Tubulin was used as a loading control.
(4.20 MB TIF)
