# Supplementary figures and images for: IKKα and IKKβ Regulation of DNA Damage-Induced Cleavage of Huntingtin
Source: PLoS One. 2009 Jun 2;4(6):e5768. doi: 10.1371/journal.pone.0005768 (PMC2685016; doi:10.1371/journal.pone.0005768)

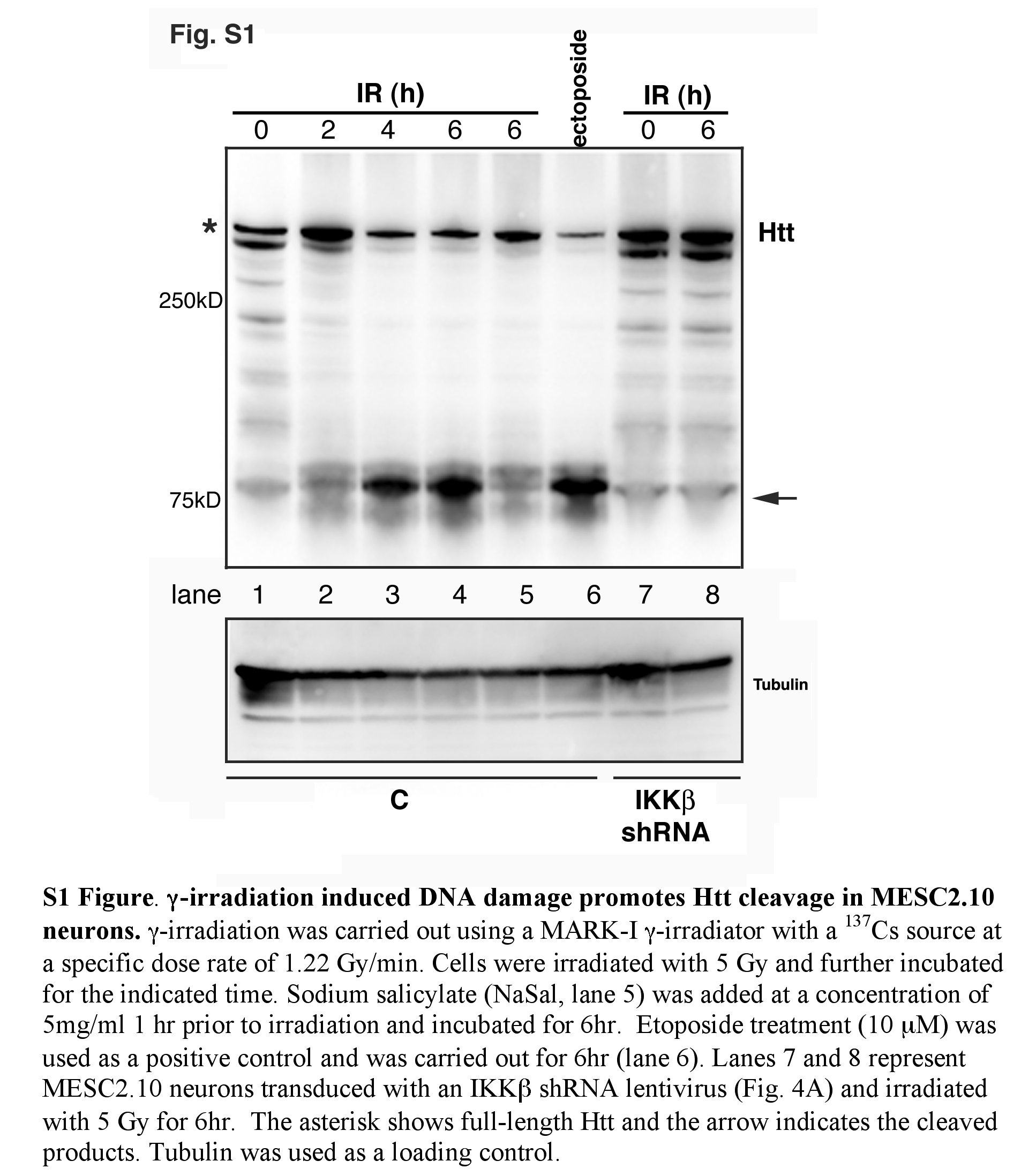

Supplement: Figure S1 — γ-irradiation induced DNA damage promotes Htt cleavage in MESC2.10 neurons. γ-irradiation was carried out using a MARK-I γ-irradiator with a 137Cs source at a specific dose rate of 1.22 Gy/min. Cells were irradiated with 5 Gy and further incubated for the indicated time. Sodium salicylate (NaSal, lane 5) was added at a concentration of 5 mg/ml 1 hr prior to irradiation and incubated for 6 hr. Etoposide treatment (10 µM) was used as a positive control and was carried out for 6 hr (lane 6). Lanes 7 and 8 represent MESC2.10 neurons transduced with an IKKβ shRNA lentivirus (Fig. 4A) and irradiated with 5 Gy for 6 hr. The asterisk shows full-length Htt and the arrow indicates the cleaved products. Tubulin was used as a loading control. (4.20 MB TIF) [file pone.0005768.s001.tif]
